# The value of global collaborations

**DOI:** 10.1002/acm2.14334

**Published:** 2024-03-24

**Authors:** Chris Trauernicht, Laurence Court

**Affiliations:** ^1^ Tygerberg Hospital and Stellenbosch University Cape Town South Africa; ^2^ Department of Radiation Physics University of Texas MD Anderson Cancer Center Houston Texas USA

Dear Editor,

We would like to review our experience of a successful global collaboration between institutions in the United States, South Africa, and several other countries. Specifically, after working together for 8 years, we would like to share this experience and encourage others to initiate similar collaborations towards reducing inequities in health care across the world.

We first met at a training course organized by the International Atomic Energy Agency (IAEA) in 2014. A team from MD Anderson then visited South Africa to propose the Radiation Planning Assistant (RPA) project, and make sure it isn't just a ‘cute physics project.’ We then started this project in 2016, with the goal of creating a tool for automated contouring and radiotherapy treatment planning, focused on the needs of clinics in low‐ and middle‐income countries (LMICs). We were very fortunate in our submission for funding from the U.S. National Cancer Institute (NCI, with Drs. Beth Beadle and Hannah Simonds), and later built on this, attracting additional funding from the Wellcome Trust (with Ajay Aggarwal of Guy's and St. Thomas’ Hospital in London, UK), Varian Medical Systems, and others. There have been many changes since then, with changes in position and institution, but most of us have continued to work together.

There have also been many changes in technology, especially in auto‐contouring which started with multi‐atlas based deformable image registration, but quickly changed to deep learning. We also changed the overall architecture of the RPA, which started as a stand‐alone tool, and is now a web‐based tool. Institutional support has also changed dramatically, with MD Anderson Cancer Center now aiming to offer this tool to clinics in LMICs for zero cost (RPA.mdanderson.org). It received regulatory approval from the U.S. Food and Drug Administration (FDA) in 2023, and is going live in South Africa in the first quarter of 2024. We hope to expand these efforts to other countries in the coming months and years. This has been quite a long path, and the route to eventual success has not always been clear, but we are finally about to start treating patients in South Africa.

Also, although our main goal is to support clinical teams, enabling more patients to be treated with high‐quality radiotherapy, we can celebrate many other achievements. These include more than 60 manuscripts, including many in the Journal of Applied and Clinical Medical Physics (JACMP). More than 15 trainees have contributed to either the current or future versions of the RPA, leading to the award of more than 13 PhDs (with more to come). On top of these obvious metrics, there are many intangible results—including the fun and challenges of working and growing together as collaborators and as a team.

The projects that we have worked on together have been very practical and, we believe, will eventually have an impact on cancer patients in LMICs. We would like to encourage our physics colleagues to engage in similar collaborations. The lack of access to radiotherapy for cancer patients in LMICs is very well documented, and we all have a huge opportunity to make a difference. Global collaborations between teams in high‐income and low‐ and middle‐income countries are a key contributor to making a difference to cancer patients across the world.

In summary, we have written this letter to thank the JACMP for publishing many of our papers, and to encourage others to collaborate with our international colleagues to try to address global inequities in access to cancer care. Persistence and collaboration can lead to a better world.

## CONFLICT OF INTEREST STATEMENT

The author declares no conflicts of interest.

